# Concentrations and health risks of selected elements in leafy vegetables: a comparison between roadside open-air markets and large stores in Johannesburg, South Africa

**DOI:** 10.1007/s10661-023-12283-6

**Published:** 2024-01-18

**Authors:** Sutapa Adhikari, Madeleen Struwig

**Affiliations:** Unit for Environmental Sciences and Management, North-West University, Private Bag X2046, Mmabatho 2790, South Africa

**Keywords:** Arsenic, Cadmium, Contamination, Deposition, Nickel, Pollution

## Abstract

This study compared concentrations and health risks of selected elements (Al, As, Cd, Co, Cr, Cu, Hg, Ni, Pb, Zn) in leafy vegetables (coriander, lettuce, mint, spring onion, swiss chard) from roadside open-air markets (OM) and large stores (supermarkets: SM, vegetable markets: VM) in Johannesburg, South Africa. Along with washed leaves (OMW, SMW, VMW), unwashed OM leaves (OMUW) were assessed to investigate the contribution of deposition. The findings revealed that OMUW leaves had the highest concentrations of all elements. Furthermore, compared with washed leaves, OMUW leaves showed significantly higher (*p* < 0.05) mean concentrations of Cd, Hg, Ni and Pb, elements that typify the composition of polluted urban air. Bi- and multivariate analysis indicated anthropogenic origin of most elements in OM leaves and several in SMW and VMW leaves. Although only OMUW leaves exhibited hazard quotient above the safe threshold of 1 for Cd, Cr, As and Ni, hazard index exceeded this safe limit in all samples (OMUW (11.77) > OMW (1.83) > SMW (1.29) > VMW (1.01)). Determined cancer risk for Cd and As was greater than 1 × 10^−6^ in both washed and unwashed leaves, and the greatest cancer risk was estimated for OM leaves. Thorough washing of OM vegetables with water reduced non-carcinogenic risk (84%) and cancer risk (74‒87%) markedly. In conclusion, residents primarily relying on open-air markets for their regular leafy vegetable supplies might face far more severe lifelong health implications compared to customers of large stores in Johannesburg.

## Introduction

Leafy vegetables are globally popular as affordable sources of carbohydrates, proteins, vitamins, essential minerals and trace elements in both traditional meals and healthy diets (Mobeen et al., 2021; Shayanowako et al., 2021). World Health Organization (WHO) recommends dietary intake of at least 400 to 500 g of fruits and vegetables per day, including leafy greens, to prevent multiple chronic diseases (WHO, 2003). However, accumulation of hazardous substances in vegetables threatens food safety and the well-being of consumers. For example, some metals and metalloids are hazardous to human health at low doses (e.g. arsenic (As), cadmium (Cd), lead (Pb) and mercury (Hg)), while others are beneficial in trace quantities but can be toxic in higher concentrations, such as chromium (Cr), cobalt (Co), copper (Cu), nickel (Ni) and zinc (Zn) (Lokeshappa et al., 2012; Ssenku et al., 2023). The adverse effects of excess intake of these elements on human health could be diverse, including replacement of structural components of biological molecules, interruption of functions of essential minerals, accumulation in vital organs and carcinogenesis (Alam et al., 2018; Mobeen et al., 2021).

Besides natural availability, anthropogenic factors such as proximity to pollution sources, wastewater irrigation, application of low-grade metal-rich agrochemicals, and ignorance and unaffordability to implement sustainable farming practices increase accumulation possibilities of elements in cultivated crops, especially in polluted urban and peri-urban regions in developing countries. Several studies have documented elevated accumulation of a wide variety of elements in leafy vegetables sold in urban markets in developing countries. For example, Bangladesh (Sultana et al., 2022), Botswana (Bati et al., 2017), Democratic Republic of Congo (Nuapia et al., 2018), Ethiopia (Eliku & Leta, 2016; Gezahegn et al., 2017), India (Lokeshappa et al., 2012), Iran (Khezerlou et al., 2021), Nigeria (Adedokun et al., 2016; Shuaibu et al., 2013), Pakistan (Khan et al., 2020), South Africa (Genthe et al., 2018; Gupta et al., 2018; Nuapia et al., 2018) and Sri Lanka (Kananke et al., 2014). Though previously overlooked, adverse effects of hazardous deposition on the quality of leafy vegetables have been recognized and discussed worldwide in recent years (Adhikari et al., 2022; Alam et al., 2018; Augustsson et al., 2023; Isiuku & Enyoh, 2020; Nabulo et al., 2012). In this case, health implications could be serious if leafy vegetables are not washed well before food preparation. Therefore, urban populations, heavily reliant on markets to fulfil their daily vegetable needs, face substantial health risks due to prolonged consumption of vegetables exposed to hazardous substances released by human activities.

Similar to many other big cities of the world, the city of Johannesburg and its neighbourhoods in South Africa have been experiencing rapid population growth and expansion in urban development (Mathee & Von Schirnding, 2003). This industrial city is located at the centre of a gold mining belt in the Witwatersrand Basin (Laker, 2023). Industries, gold mines and tailings cause widescale pollution of air, soil, surface waters and aquifers in the region (Chetty et al., 2021; Kamunda et al., 2016; Laker, 2023; Mathee et al., 2018; Rösner & Van Schalkwyk, 1999; Tibane & Mamba, 2022). The cosmopolitan population in the region depends on different types of markets for their vegetable supplies based on affordability, preferences and availability of local and exotic vegetables. However, comparable to other polluted cities in South Africa, the current knowledge of hazardous element contamination of market vegetables in Johannesburg is based on very limited data. Among various types of markets, open-air markets are one of the major vegetable distribution points in many big cities in developing countries, including South Africa (Alam et al., 2018; Nuapia et al., 2018). However, despite striking differences in market/selling conditions and consumer groups between roadside open-air markets and large stores, elemental contents of leafy vegetables from these two types of markets in Johannesburg have not been compared thus far. Therefore, we carried out this study to compare leafy vegetables from roadside open-air markets and large stores (supermarkets and vegetable markets) in Johannesburg in terms of (1) concentrations of selected elements, (2) potential sources of elements and (3) associated health risks for consumers.

## Materials and methods

### Collection and preparation of leafy vegetables

The following five leafy vegetables, coriander (*Coriandrum sativum*, abbreviated as ‘Cor’, *n* = 30), lettuce (*Lactuca sativa*, ‘Let’, *n* = 30), mint (*Mentha* sp., ‘Min’, *n* = 30), spring onion (*Allium fistulosum*, ‘Spr’, *n* = 30) and swiss chard (*Beta vulgaris* L. var. *cicla*, ‘Swi’, *n* = 30) were procured from ten randomly selected shops under roadside open-air market (OM), supermarket (SM) and vegetable market (VM) category in Johannesburg. These five leafy vegetables were selected based on their availability in both open-air markets and large stores across Johannesburg. All evaluated vegetables were produced in South Africa. Samples of each leafy vegetable, procured from ten shops within each market category, were combined to form a composite sample. Because open-air markets in Johannesburg operate in congested areas and within 5–10 m from traffic, half of the OM samples were not washed and used as unwashed materials (denoted as ‘UW’). These samples were numbered CorUW1, LetUW2, MinUW3, SprUW4 and SwiUW5 under the group OMUW. The rest of the OM samples were washed (W) and numbered CorW6, LetW7, MinW8, SprW9 and SwiW10 under the group OMW. On the contrary, since supermarkets and vegetable markets present low chances of dust exposure under in-store conditions and sell pre-cleaned vegetables, all samples acquired from these large stores were washed. These samples were numbered CorW11, LetW12, MinW13, SprW14 and SwiW15 under SMW, and CorW16, LetW17, MinW18, SprW19 and SwiW20 under VMW. Leafy vegetables were washed thoroughly with running tap water to simulate generally followed vegetable preparation procedures in households (Augustsson et al., 2023).

### Sample digestion and analysis

Washed and unwashed leaves were chopped finely and dried in an oven at 35 °C for 48 h. Leaves were then milled to powder, and the EPA 3051A microwave method was applied to digest the materials. From each powdered sample, 200 mg (dry wt) was taken in individual Teflon tubes. To each tube, 9 ml of 65% nitric acid (HNO_3_) and 3 ml of 32% hydrochloric acid (HCl) (HNO_3_–HCl, 3:1, v/v) were added. The tubes were then sealed and placed in a high-performance microwave digestion system (Milestone, Ethos UP, Maxi 44) for 20 min until the system reached 200 °C. This state was maintained for 15 min, after which the tubes were allowed to cool. The volume in each tube was adjusted to 50 ml. In leaf samples, total concentration of each of the following metals, Al, Cd, Co, Cr, Cu, Hg, Ni, Pb and Zn, and the only metalloid, As, was determined with ICP-MS (Agilent). Elemental concentrations in leafy vegetables were expressed in dry weight (mg kg^−1^ dry wt).

### Quality control

The ICP-MS was calibrated, and precautionary measures were followed. Accuracy was ensured by repeating the analysis. The stability of the ICP-MS was checked after every six samples by analyzing the standard solution and sample blanks. All the reagents and solvents were of analytical grade (Merck, Germany). Ultrapure deionized water from a Milli-Q analytical reagent-grade water purification system was used throughout the analysis processes. Sample introduction was achieved via a Micromist-type nebulizer (which reduces matrix interferences and achieves a more robust plasma) and standard quarts spray chamber. The instrument was optimized using a solution containing Li, Y, Ce and Tl (1 ppb) for standard low-oxide/low interference levels (≤ 1.5%), at the same time maintaining high sensitivity across the mass range. Typical instrumental conditions are summarized in Table 1. To obtain quantitative results, the instrument was calibrated using ULTRASPEC® certified custom mixed multi-element stock standard (De Bruyn Spectroscopic Solutions, South Africa) solutions containing all the elements of interest. The analysis was conducted under hygienic laboratory conditions and adhering to recommended protocols. Quality control standards were employed to ensure the correct criteria were met for all analyzed samples.

**Table 1:** Instrument parameters

Parameter	Value
Forward power	1550 W
Plasma gas flow	15 L/min
Nebulizer gas flow	1.2 L/min
Sampling depth	8 mm
Spray chamber temperature	2 °C

### Data analysis

Data showed non-normal distributions (Shapiro–Wilk test, *p* < 0.05). Kruskal–Wallis test and pairwise comparisons were performed to assess if the mean levels of individual and collectively all elements differed significantly (1) among the five leafy vegetables within each group (OMUW, OMW, SMW and VMW) and (2) among the four groups (combining five species under each group). Within each of the four vegetable groups, relationships between elements were determined using Spearman’s correlation coefficient. Principal component analysis (PCA) was conducted using the direct oblimin method and Kaiser normalization to investigate correlation driven associations among elements within each group. Correlation matrices and PCA were employed to identify the sources of elements in vegetables and, hence, assess the contributions of anthropogenic factors toward accumulation of elements of concern. Statistical significance was considered at *p* value less than 0.05. Statistical analysis was carried out using SPSS software (IBM version 28.0).

### Human health risk assessment

#### Non-carcinogenic risk

The following indices were used to assess non-carcinogenic risks linked to evaluated elements: Estimated Daily Intake (EDI, mg kg^−1^ bw day^−1^), Hazard Quotient (HQ) and Hazard Index (HI). EDI of each of the ten elements (Al, As, Cd, Co, Cr, Cu, Hg, Ni, Pb and Zn) was calculated based on the mean concentration combining five leafy vegetables in each group (Eq. 1). 

1 $$EDI=\frac{C\times Cf\times DC}{BW}$$

In Eq. (1), ‘C’ indicates the mean concentration of the element (mg kg^‒1^ dry wt), ‘Cf’ is fresh to dry weight conversion factor (0.085), ‘DC’ is the daily vegetable consumption quantity (0.0989 kg day^−1^) (Vorster et al., 2013) and ‘BW’ is the average body weight of an adult (70 kg, according to WHO).

To assess the potential toxicological effects of each element, HQ of As, Cd, Co, Cr, Cu, Ni, Pb and Zn were calculated using Eq. (2). Determined HQ of eight elements were then summed to estimate Hazard Index (HI) for each of the four groups, i.e. OMUW, OMW, SMW and VMW (Eq. 3). HI predicts non-carcinogenic risk associated with simultaneous dietary exposure to all these elements over a lifetime.

2 $$HQ=\frac{EDI}{RfD}$$

3 $$HI= HQAs+HQCd+HQCo+HQCr+HQCu+HQNi+HQPb+HQZn$$

In Eq. (2), the Reference Dose (RfD) represents the maximum acceptable oral dose of a toxic substance. The oral RfD for As, Cd, Co, Cr, Cu, Ni, Pb and Zn were 0.0003, 0.001, 0.03, 0.003, 0.04, 0.02, 0.0035, and 0.3 mg kg^‒1^ day^‒1^, respectively (Finley et al., 2012; Taylor et al., 2022; Voica et al., 2021). When HQ and HI values surpass the safe threshold of 1, it signifies a high likelihood of non-carcinogenic risks to consumers. Conversely, these risks may be inconsequential when the values are below or equal to 1.

Al and Hg were excluded from HQ and HI assessments due to the unavailability of corresponding RfD values (IRIS, 1995a; USEPA, 2006). Instead, the Estimated Weekly Intake (EWI) was calculated using the respective EDI (EWI = EDI × 7, assuming seven days a week). These EWI values were then compared with the Tolerable Weekly Intake (TWI) suggested by the European Food Safety Authority (EFSA) and the Provisional Tolerable Weekly Intake (PTWI) recommended by the Joint FAO (Food and Agricultural Organization)/WHO Expert Committee on Food Additives (JECFA) to determine potential adverse effects of these two elements on consumer health (Brzezińska-Rojek et al., 2022; Hardisson et al., 2017).

#### Cancer risk

Cancer risk (CR) was estimated for As, Cd and Pb, elements that are known to induce cancer through oral exposure (Carver & Gallichio, 2017; Nunes & Otero, 2017; Cai et al., 2019). CR was calculated by multiplying EDI with the corresponding slope factor (SF) of each element (Eq. 4). The SF is defined as an upper bound limit indicating the increased risk of cancer from lifetime exposure to a substance. The oral SF for As, Cd and Pb were 1.5, 0.38 and 0.0085 mg kg^‒1^ day^‒1^, respectively (Can et al., 2020; IRIS, 1995b). Cancer risk below 1 × 10^‒6^ (≤ 1 in 1,000,000 individuals exposed similarly) could be least concern for increased cancer risk, whereas values ranging between 1 × 10^‒6^ and 1 × 10^‒4^ and above might be of concern (USEPA, 2005).

4 $$CR=EDI\times SF$$

## Results and discussion

### Concentrations of elements in leafy vegetables

Combining the data from five vegetables across the four groups (OMUW, OMW, SMW and VMW), mean concentrations of elements (mg kg^−1^ dry wt) varied within the following ranges: Al (318.11–1413.92), As (0.36–2.56), Cd (2.10–51.47), Co (2.19–6.37), Cr (7.79–74.31), Cu (34.30–46.52), Hg (0.12–2.45), Ni (8.00–198.71), Pb (0.026–0.037) and Zn (165.23–238.64) (Table 2). Mean levels of ten elements decreased in the following order: Al > Zn > Ni > Cr > Cd > Cu > Co > As > Hg > Pb in OMUW leaves and Al > Zn > Cu > Cr > Ni > Cd > Co > As > Hg > Pb in OMW leaves. The following descending orders were observed for SMW leaves: Al > Zn > Cu > Ni > Cr > Cd > Co > As > Hg > Pb and VMW leaves: Al > Zn > Cu > Ni > Cr > Co > Cd > As > Hg > Pb (Table 2). In all four groups, the highest and the lowest concentration means were observed for Al and Pb, respectively. Among all samples, OMUW leaves had the highest mean concentrations of all assessed elements (Table 2). In particular, multiple times higher concentrations were observed for Cd (7, 20 and 24 times than those of OMW, SMW and VMW), Cr (6, 5 and 9 times than those of OMW, SMW and VMW), Hg (12, 19 and 17 times than those of OMW, SMW and VMW) and Ni (24, 14 and 15 times than those of OMW, SMW and VMW). It appears that deposition led to contamination of OMUW leafy vegetables with highly toxic elements. It was further observed that irrespective of market type, coriander, mint and swiss chard had relatively higher mean levels of most elements compared to lettuce and spring onion (Table 2). Among all samples, unwashed coriander leaves showed far higher concentrations of all elements.

**Table 2:** Concentrations of evaluated elements (mg kg^−1^ dry wt) in leafy vegetables from markets in Johannesburg

Market	Sample number	Al	As	Cd	Co	Cr	Cu	Hg	Ni	Pb	Zn
Open-air markets (unwashed, OMUW)	CorUW1	3571	11.5	191.90	20.26	276	49.71	9.76	822.20	0.0487	190.60
	LetUW2	154.80	0.22	31.55	1.45	66.65	12.65	1.68	129.60	0.0296	125.10
	MinUW3	1250	0.7213	24.29	0.8891	9.41	64.28	0.4973	26.94	0.0422	477.2
	SprUW4	417.80	0.3327	5.71	1.29	10.53	20.79	0.176	8.044	0.0285	121.4
	SwiUW5	1676	0.5761	3.91	7.97	23.85	85.19	0.1332	7.572	0.0387	278.90
	Mean ± SD	1413.92 ± 1353.35	2.56 ± 4.99	51.47 ± 79.39	6.37 ± 8.29	74.31 ± 115.41	46.52 ± 30.12	2.45 ± 4.13	198.71 ± 352.21	0.0376 ± 0.008	238.64 ± 147.88
Open-air markets (washed, OMW)	CorW6	3146	1.31	6.61	2.61	19.82	38.18	0.2401	10.5	0.0311	147.30
	LetW7	56.22	0.2117	1.39	1.11	3.92	7.76	0.1006	4.529	0.0319	81.86
	MinW8	566.30	0.5105	17.24	1.64	8.87	64.48	0.2514	11.52	0.0331	209.40
	SprW9	168.50	0.3095	3.99	1.41	4.93	14.74	0.2129	5.923	0.0344	128.50
	SwiW10	284	0.2774	4.97	7.87	8.99	70.03	0.201	6.789	0.0347	259.10
	Mean ± SD	844.20 ± 1300.66	0.6321 ± 0.43	6.84 ± 6.11	2.93 ± 2.81	12.28 ± 9.02	39.03 ± 28.18	0.2012 ± 0.05	8.00 ± 2.96	0.0330 ± 0.001	165.23 ± 69.61
Supermarkets (washed, SMW)	CorW11	270.80	0.3174	3.68	0.8298	5.83	39.18	0.1281	18.11	0.0343	216.70
	LetW12	48.76	0.2248	2.55	2.84	5.47	32.14	0.1327	13.14	0.02657	223.90
	MinW13	775	0.5375	2.09	1.10	43.65	35.03	0.1586	16.87	0.02775	132.90
	SprW14	216.70	0.3179	2.11	1.26	7.21	21.86	0.1042	12.33	0.0242	150.60
	SwiW15	279.30	0.4044	2.04	4.91	7.07	34.41	0.1069	10.41	0.03084	394.90
	Mean ± SD	318.11 ± 271.72	0.3604 ± 0.11	2.50 ± 0.69	2.19 ± 1.71	13.85 ± 16.67	32.52 ± 6.48	0.1261 ± 0.02	14.17 ± 3.21	0.0287 ± 0.003	223.80 ± 103.61
Vegetable markets (washed, VMW)	CorW16	730.60	0.3448	1.95	1.18	5.78	34.30	0.1315	18.06	0.02662	249.30
	LetW17	397.40	0.2829	1.32	3.44	4.56	23.94	0.1749	6.69	0.01853	195.80
	MinW18	176	0.4263	2.37	1.49	12.57	87.60	0.1582	24.67	0.03181	224.30
	SprW19	500.30	0.4035	1.91	1.36	10.51	14.81	0.1163	7.06	0.02087	213.40
	SwiW20	517.50	0.3607	2.95	7.11	5.52	45.56	0.1142	7.10	0.03255	163.80
	Mean ± SD	464.36 ± 201.64	0.3636 ± 0.05	2.10 ± 0.60	2.92 ± 2.51	7.79 ± 3.52	41.24 ± 28.34	0.1390 ± 0.02	12.71 ± 8.23	0.0260 ± 0.006	209.32 ± 31.99
FAO/WHO permissible level		12‒71^a^	0.2^b^	0.2^c^	50^d^	2.3^d^	73.3^d^	0.5^e^	66.9^d^	0.3^d^	99.4^d^

^a^Nuapia et al., 2018

^b^CAC, 2014

^c^Sultana et al., 2022

^d^Rahmdel et al., 2018

^e^Ssenku et al., 2023

**Fig. 1:**
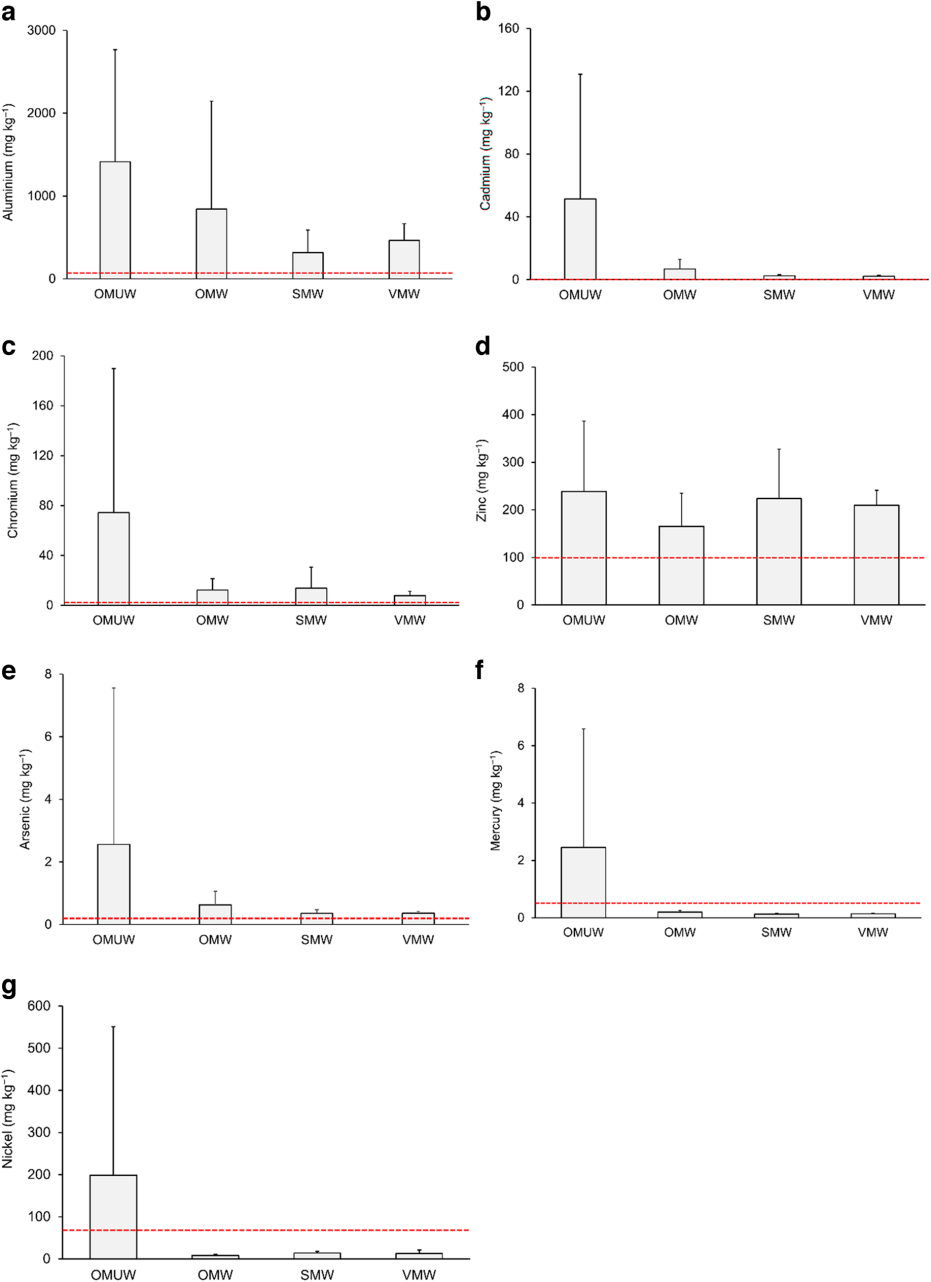
Elements for which mean concentrations exceeded permissible levels (dashed lines) in leafy vegetables, **a** aluminium, **b** cadmium, **c** chromium, **d** zinc, **e** arsenic, **f** mercury and **g** nickel. OM open-air markets, SM supermarkets, VM vegetable markets, UW unwashed leaves, W washed leaves

Mean concentrations of Co, Cu and Pb remained below FAO/WHO permissible levels of 50 mg kg^‒1^, 73.3 mg kg^‒1^ and 0.3 mg kg^‒1^, respectively, in all groups (Table 2). Conversely, mean levels of Al, Cd, Cr and Zn surpassed corresponding FAO/WHO maximum permissible levels under all four groups (Table 2, Fig. 1a–d). Mean values of As exceeded the threshold of 0.2 mg kg^‒1^ in both unwashed and washed OM leaves (Table 2, Fig. 1e). Of all four groups, mean concentrations of Hg (Table 2, Fig. 1f) and Ni (Table 2, Fig. 1g) exceeded their respective standards (Hg: 0.5 mg kg^‒1^; Ni: 66.9 mg kg^‒1^) only in OMUW leaves. In South Africa, maximum allowable levels of Cd and Pb were available for leafy vegetables, which aligns with FAO/WHO standards (Department of Health, 2018). Department of Health (2018) established the food-grade limit of total Hg at 0.1 mg kg^‒1^, and mean Hg concentrations in leafy vegetables from all markets surpassed this threshold.

Kruskal–Wallis test indicated non-significant differences in mean concentrations of individual elements among the five species within and among the groups. However, considering all elements, unwashed coriander from open-air markets showed significantly higher accumulation compared to washed lettuce from OM (*p* = 0.009) and SM (*p* = 0.038), and washed spring onion from OM (*p* = 0.029), SM (*p* = 0.034) and VM (*p* = 0.037). Further comparison among leafy vegetable groups revealed that OMUW leaves (combining five vegetables) had significantly higher mean concentrations of Ni (*p* = 0.042) compared to OMW leaves, Cd (*p* = 0.013) and Hg (*p* = 0.008) than SMW leaves, and Cd (*p* = 0.001), Hg (*p* = 0.028) and Pb (*p* = 0.026) than VMW leaves.

The observed similarities in element concentration trends in vegetables from supermarkets and vegetable markets, and their differences from open-air market vegetables, possibly relate to the fact that bigger agricultural farms supply supermarkets and vegetable markets, whereas vegetables sold in open-air markets mainly come from smallholder farmers. Understandably, exposure to pollutants during cultivation, post-harvest processing, storage and transportation varies depending on the suppliers, as well as according to market and selling conditions (Rahmdel et al., 2018). Furthermore, quality assurance practices implemented by large farms and stores such as washing and packaging of vegetables minimize surface particle quantities (Bati et al., 2017; Nabiha & Wulandari, 2020).

In this regard, the impact of market-related factors (types and locations) has been recognized on a global scale (Alam et al., 2018; Isiuku & Enyoh, 2020; Nabiha & Wulandari, 2020). This fact was reflected by elevated levels of elements, especially Cd, Ni, Hg and Pb in leafy vegetables from roadside open-air markets compared to larger stores in Johannesburg. Hence, this study confirms that atmospheric deposition results in distinct element accumulation trends in leafy vegetables from open-air markets than that of in-store conditions. Therefore, in polluted urban environments, the influence of market type should be duly considered. This further emphasizes the importance of considering locality-specific sources and exposure pathways of various elements to manage contamination of cultivated vegetables.

In terms of comparison between markets, no records were obtained for the vegetables assessed in this study. However, Nabiha and Wulandari (2020) noted far greater quantities of Pb in kale from traditional markets than a supermarket in Indonesia. On the contrary, in Botswana, vegetables from supermarkets showed greater concentrations of a number of elements compared to the ones sold by street vendors (Bati et al., 2017). Another study by Isiuku and Enyoh (2020) noted differences in concentrations of several elements in four different leafy vegetables from Nigerian markets. In South Africa, contrasting the present study, previous investigations documented relatively lower levels of elements in various other market vegetables and fruits from Durban (Gupta et al., 2018), Olifants River catchment area (Genthe et al., 2018), and roadside open markets in Johannesburg (Nuapia et al., 2018).

No record was obtained on elemental contents of unwashed leaves of selected species to compare with the findings of this study. However, with regard to washed leaves, elemental contents were largely consistent with data for these five leafy vegetables from markets in other regions. Khezerlou et al. (2021) reported similar concentrations of Cd and Cr, lower Cu, Ni and Zn, and higher levels of As and Pb in lettuce from markets in Iran. A study by Sultana et al. (2022) noted comparable concentrations of Cr and Cu, lower Cd, Ni and Zn, but higher Pb concentrations in mint and coriander from markets in Bangladesh. Levels of Al observed in coriander leaves in this study corresponded to those documented for this popular herb from industrial localities in India (Mandal & Kaur, 2020) and Iran (Ghasemidehkordi et al., 2018). Shakya and Khwaounjoo (2013) found far higher levels of Cd and Pb in coriander from markets in Nepal. Spring onions from a copper-tungsten mining site in Korea showed lower concentrations of Cu, higher levels of Cd and Pb, and similar concentrations of Zn (Jung, 2008).

Similar to this study, excess accumulation of Al, Cd, Cr, Cu, Pb and Zn has been recorded in cultivated coriander from polluted industrial, urban and peri-urban regions in Pakistan (Farooq et al., 2008), India (Lokeshappa et al., 2012) and Qatar (Alsafran et al., 2021). Likewise, considerably high levels of As, Cr, Cu, Pb and Zn were noted in mint from urban markets in Bangladesh (Sultana et al., 2022), farms in Iraq (Abdulazeez & Aziz, 2014), chard irrigated with wastewater (Eliku & Leta, 2016) and cultivated near the capital city of Ethiopia (Gezahegn et al., 2017). Several urban-based studies have documented high as well as low concentrations of hazardous elements in lettuce (Baldantoni et al., 2016; Shuaibu et al., 2013) and spring onion (Jung, 2008; Khan et al., 2020) as found in this Johannesburg study. This study therefore supports the fact that species and genotype driven selective uptake primarily regulate accumulation of beneficial and toxic elements in plant parts (Baldantoni et al., 2016; Moyo et al., 2020; Shahid et al., 2017). We also report noteworthy airborne dust accumulation by coriander leaves. Contrasting adequate reports on hazardous element accumulation in leaves of coriander, there is a lack of information on its dust accumulation and retention properties, which emphasizes the need for further exploration.

### Source identification of elements in leafy vegetables

Spearman’s correlation coefficient values (*R*_s_) indicated significant relationships between multiple elements within all groups. A strong correlation is indicated by a value of *R*_s_ ranging between 0.9 and 1. Under OMUW group, positive correlations were observed between Al–As, As–Pb, Co–Cr (*R*_s_ = 0.9, *p* = 0.037), Cd–Hg and Cd–Ni (*R*_s_ = 1, *p* = 0.001). In OMW leaves, Al was significantly and positively correlated with As, Cd, Cr and Ni (*R*_s_ = 0.9, *p* = 0.037). Further significant positive correlations were indicated between As–Hg, Co–Cr, Co–Cu, Hg–Ni (R_s_ = 0.9, *p* = 0.037), Cd–Hg, Cd–Ni and Zn–Cu (*R*_*s*_ = 1, *p* = 0.001). In the case of SMW, significant positive correlations were determined between Al–As, As–Cr and Cu–Pb (*R*_*s*_ = 0.9, *p* = 0.037). A significant negative correlation was observed between Co–Ni (*R*_*s*_ = ‒0.9, *p* = 0.037). Under VMW group, significant positive correlations were indicated between As–Cr (*R*_*s*_ = 0.9, *p* = 0.037) and Cd–Pb (*R*_*s*_ = 1, *p* = 0.001), whereas a significant negative relation was indicated between Co–Zn (*R*_*s*_ = ‒0.9, *p* = 0.037). In this case, a strong positive correlation between elements is indicative of their shared sources, while different sources could be assumed for elements correlated negatively.

Correlation-based PCA further indicated associations among elements in evaluated market leafy vegetables (Table 3). For OMUW leaves, the first component (PC1) explained 72.76% of the total data variance. This component had high positive loadings of Al, As, Cd, Co, Cr, Hg, Ni and Pb. The second component, PC2, had high positive loadings of Cu, Pb and Zn and contributed 22. 87% of the total data variance. For OMW, Al, As and Cr had high positive loadings and a negative loading of Pb on PC1. Co, Cu, Pb and Zn had greater positive loadings on PC2. The two components contributed 48.18% and 33.23% of the total data variance, respectively. Under SMW group, large loadings of Al, As, Cr and Hg were observed on PC1, whereas on PC2, Cd, Cu, Ni and Pb had high positive loadings. Data variance contributions from PC1 and PC2 were 44.54% and 28.76%, respectively. In VMW vegetables, PC1 contributed 41.15% of the total data variance. This component showed high positive loadings of Cd, Cu, Ni and Pb. Presenting 30.31% of total data variance, PC2 showed a high positive loading of Co and high negative loadings of Ni and Zn. Overall, PCA outcome supported correlation results explained in the previous sections.

**Table 3:** Direct oblimin rotated and Kaiser normalized component loadings of elements accumulated in market leafy vegetables

	OMUW		OMW		SMW		VMW	
	PC 1	PC 2	PC 1	PC 2	PC 1	PC 2	PC 1	PC 2
Al	0.883	0.375	1.000	0.13	0.952	− 0.050	− 0.063	− 0.331
As	0.997	− 0.019	0.959	− 0.049	0.974	0.035	0.221	− 0.112
Cd	0.993	− 0.085	− 0.131	− 0.123	− 0.496	0.753	0.732	0.379
Co	0.947	0.098	0.087	1.034	− 0.008	0.040	0.304	0.854
Cr	0.996	− 0.171	0.961	0.235	0.943	− 0.098	− 0.015	− 0.177
Cu	0.048	0.937	0.022	0.696	0.273	0.944	0.824	− 0.071
Hg	0.997	− 0.138	0.27	0.215	0.631	0.241	− 0.038	− 0.132
Ni	0.999	− 0.137	0.415	− 0.021	0.211	0.521	0.677	− 0.672
Pb	0.716	0.629	− 0.698	0.543	− 0.025	0.977	0.975	0.087
Zn	− 0.220	0.913	− 0.096	0.825	− 0.13	0.384	0.032	− 1.011
Explained variance (%)	72.76	22.87	48.18	33.23	44.54	28.76	41.15	30.31

*n* = 5 in each market group

OM; open-air markets, SM; supermarkets, VM; vegetable markets, UW; unwashed leaves, W; washed leaves

The common sources of Al, As, Pb, Cd, Hg and Ni in unwashed leafy vegetables could be mostly anthropogenic, including automobile exhaust, road dust, emissions from industries, mines and coal-based power stations (Chetty et al., 2021; Huang et al., 2017; Li et al., 2017; Sezgin et al., 2004; Zgłobicki & Telecka, 2021). Transportation of uncovered, unpacked or improperly packed vegetables, sprinkling water over displayed leaves and the absence of barriers such as buildings or vegetation between shops and roads in most localities probably enhance deposition on leafy vegetables in open-air markets in Johannesburg (Alam et al., 2018; Khan et al., 2020).

It is difficult to remove particles from leaf surfaces by washing with water because different leaf morphological features, especially surface microstructures, retain large proportions of deposited particles (Adhikari et al., 2022; Augustsson et al., 2023). Additionally, possible foliar uptake of Cd, Pb, Cu, Zn, Cr, Ni, Hg and As contributes to the elemental composition of leaf tissue (Pleijel et al., 2021; Sachan & Lal, 2017; Shahid et al., 2017). Therefore, aerial deposition might be a common source of As, Cd, Cr, Hg and Ni accumulated in washed leafy vegetables from open-air markets. Conversely, primary common sources of most of the Cu, Co, Cr and Zn in washed vegetables from open-air markets could be rocks and minerals, and soil (Nuapia et al., 2018; Tibane & Mamba, 2022).

The majority of accumulated Co, Cr, Ni and As in leafy vegetables from large stores (SM and VM) could have come from the soil (Adhikari et al., 2022; Nuapia et al., 2018). Some of the stored Cu, Zn, Pb, Ni and Cd in leafy vegetables from big stores may also be attributed to cultivation practices, including waste or sludge application, overuse of farming products and irrigation with contaminated water (Genthe et al., 2018; Mathee et al., 2018; Moyo et al., 2020; Nabulo et al., 2012). This explains the indicated differences in sources of Co and Ni in SMW and Co and Zn in VMW. For Al, a major component of the Earth’s crust and soil, high concentrations in leafy vegetables from all markets may be linked to its natural abundance (ATSDR, 2008; Ghasemidehkordi et al., 2018). Moreover, in addition to suspended Al containing particles of geogenic origin, emissions from various fugitive and point sources, such as mining, agricultural activities, coal combustion, smelting and crustal dust, contribute to aerial Al levels. This is a concern in urban sites (ATSDR, 2008). Deposition of airborne Al is highly plausible in the case of leafy vegetables sold in open-air markets in Johannesburg.

Compared to national and international permissible levels, higher concentrations of Cr, Ni and As in soils of the Witwatersrand basin and Johannesburg, as well as Cu and Zn enrichment of tailings soils in the gold mining areas, have been documented (Kamunda et al., 2016; Tibane & Mamba, 2022). Although Rösner and Van Schalkwyk (1999) noted low availability of most elements in gold mine tailings in this mining region, more recent studies have indicated varying degrees of ecological and health risks associated with elemental contents of mining impacted soils (Kamunda et al., 2016; Laker, 2023; Mathee et al., 2018; Tibane & Mamba, 2022). Accumulation of Hg in edible plants around gold mining areas has been documented by Mahmud et al. (2019) in North Gorontalo Regency, Gorontalo Province. A comprehensive study of the elemental composition of deposited dust, agricultural soils and crops is therefore essential to further elucidate sources of elements accumulated in vegetables.

### Health risks of elements in leafy vegetables

Determined EDI, HQ and HI are summarized in Table 4. In OMUW group, EDI of As, Cd, Cr and Ni exceeded respective oral RfD. Subsequently, HQ of Cd (6.2542), Cr (3.0100), Ni (1.2072) and As (1.0384) violated the safe threshold of 1 in unwashed OM leaves. On the contrary, HQ of all assessed elements (As, Cd, Co, Cr, Cu, Ni, Pb, Zn) remained well under the safe limit of 1 in washed leaves from all markets, i.e. OM, SM and VM. However, for both washed and unwashed vegetables, the resultant HI was greater than 1. The highest HI was estimated for OMUW leaves (11.7749), followed by OMW (1.8319), SMW (1.2960) and VMW leaves (1.0186). Thorough washing of OMUW leaves decreased HI by nearly 84%. HI for OMUW leaves was 6, 9 and 12 orders of magnitude higher than that of OMW, SMW and VMW, respectively. Furthermore, the HI for OMW group was approximately 1.4 and 1.8 times greater than the HI estimated for SMW and VMW leafy vegetables, respectively.

**Table 4:** Estimated Daily Intake (EDI), Hazard Quotient (HQ) and Hazard Index (HI) of elements in leafy vegetables from markets in Johannesburg, South Africa

	OMUW		OMW		SMW		VMW	
	EDI	HQ	EDI	HQ	EDI	HQ	EDI	HQ
As	0.000311	1.038453	0.000076	0.256037	0.000043	0.145969	0.000044	0.147281
Cd	0.006254	6.254215	0.000831	0.831512	0.000303	0.303789	0.000255	0.255818
Co	0.000774	0.025816	0.000356	0.01187	0.000266	0.008871	0.000355	0.011834
Cr	0.009030	3.010069	0.001492	0.497404	0.001682	0.560984	0.000946	0.315558
Cu	0.005652	0.141323	0.004743	0.118586	0.003951	0.098796	0.005011	0.125278
Ni	0.024144	1.207248	0.000973	0.048656	0.001721	0.086099	0.001545	0.077264
Pb	0.000004	0.001142	0.000004	0.001005	0.000003	0.000873	0.000003	0.000792
Zn	0.028996	0.096654	0.020076	0.066922	0.027192	0.090643	0.025433	0.084779
HI	-	11.7749	-	1.8319	-	1.2960	-	1.0186

OM; open-air markets, SM; supermarkets, VM; vegetable markets, UW; unwashed leafy vegetables, W; washed leafy vegetables

**Fig. 2:**
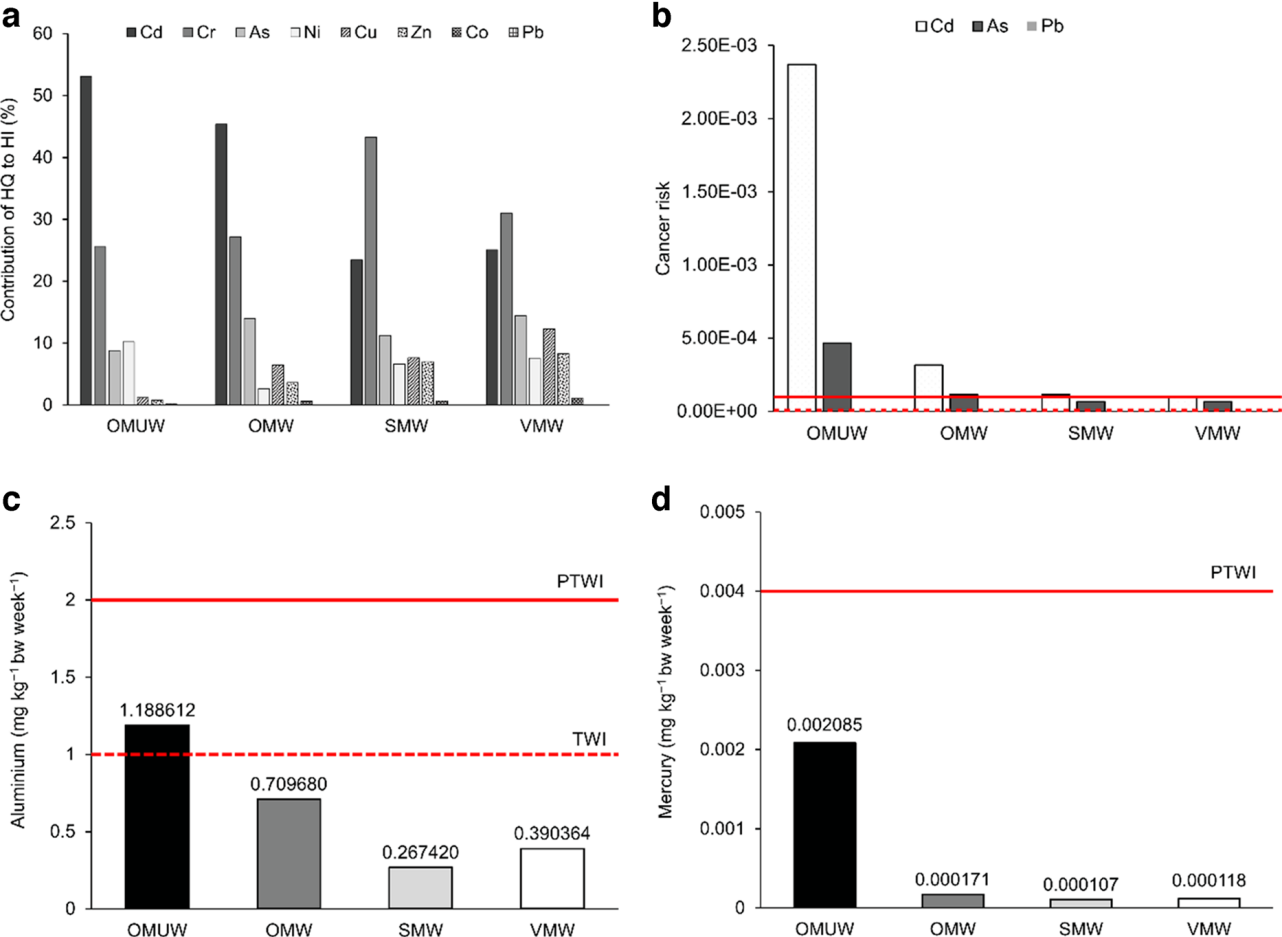
Health risks of selected elements in leafy vegetables from OM, SM and VM, **a** percentage contribution of hazard quotient (HQ) of each element to hazard index (HI), **b** cancer risk of As, Cd and Pb, **c** estimated weekly intake of aluminium and **d** estimated weekly intake of mercury. Cancer risk: dotted line, 1 × 10^‒6^; solid line, 1 × 10^‒4^. OM open-air markets, SM supermarkets, VM vegetable markets, UW unwashed leafy vegetables, W washed leafy vegetables

Evaluation of percentage (%) contribution of HQ of each element to HI further explained trends in health risks associated with market leafy vegetables from roadside open-air markets and big stores in Johannesburg (Fig. 2a). Under OMUW group, contributions of HQ of each element to HI followed the descending order of Cd (53.11%) > Cr (25.56%) > Ni (10.25%) > As (8.81%) > Cu (1.2%) > Zn (0.82%) > Co (0.21%) > Pb (0.009%). In this group, the cumulative contribution of HQ of Cd, Cr, Ni and As was nearly 98%. The OMW group showed a slightly different trend than what was observed for washed vegetables from large stores by having the largest contribution from HQ of Cd (45.38%), followed by Cr (27.15%), then As (13.97%), Cu (6.47%), Zn (3.65%), Ni (2.65%), Co (0.64%) and Pb (0.051%). In this case, HQ of Cd, Cr, As and Cu contributed the most, around 93% to HI. For both SMW (Cr (43.28%) > Cd (23.44%) > As (11.26%) > Cu (7.62%) > Zn (6.99%) > Ni (6.64%) > Co (0.68%) > Pb (0.068%)) and VMW (Cr (30.97%) > Cd (25.11%) > As (14.45%) > Cu (12.29%) > Zn (8.32%) > Ni (7.58%) > Co (1.16%) > Pb (0.077%)), the largest contributor was HQ of Cr and assessed elements followed the same order. Unlike OM groups, in both SMW and VMW groups, contributions of Cd and Cr decreased, while those of other elements increased (especially Cu and Zn), except for Co and Pb. In both of these groups, HQ values of Cr, Cd, As, Cu, Zn and Ni contributed approximately 99% to the corresponding HI. Regarding individual elements, trends in health risks varied between unwashed leaves (high risks associated with multiple individual elements) and washed leaves (no risk was predicted from any one element) (Table 4). In terms of risk from all elements (HI), leafy vegetables were largely differentiated between open-air markets (HQ of Cd as the dominant contributor to HI) and large stores (HQ of Cr was the biggest contributor). However, for both types of markets, HQ of Cd and Cr were the two main contributors to their respective HI (Fig. 2a).

Cancer risk (CR) of Cd and As was above 1 × 10^‒4^ in both OMUW (Cd: 2.38E − 03, As: 4.67E − 04) and OMW leaves (Cd: 3.15E − 04, As: 1.15E − 04) (Fig. 2b). SMW vegetables also showed CR higher than 1 × 10^‒4^ for Cd (1.15E − 04), whereas CR of As (6.56E − 05) remained between 1 × 10^‒6^ and 1 × 10^‒4^ (Fig. 2b). In the case of VMW, CR of both Cd (9.72E − 05) and As (6.62E − 05) stayed between 1 × 10^‒6^ and 1 × 10^‒4^. CR of Cd through OMUW leaves was approximately 8, 20 and 24 times higher than the CR of OMW, SMW and VMW leaves, respectively. Moreover, CR of As in OMUW leaves was nearly 4 times greater compared to OMW and 7 times higher than that of both SMW and VMW leaves. In addition, CR of Cd and As in OMW leaves was nearly 3 and 2 times higher, respectively, than the corresponding CR determined for both SMW and VMW vegetables. Washing leafy vegetables from open-air markets reduced CR linked to Cd and As by 87% and 74%, respectively. The lowest CR was determined for Pb, which was lesser than 1 × 10^‒6^ in all four groups (OMUW: 3.88E − 08 > OMW: 3.41E − 08 > SMW: 2.96E − 08 > VMW: 2.69E − 08) (Fig. 2b). This suggested that CR of Cd and As accumulated in leafy vegetables from OM, SM and VM might be of sufficient concern. On the contrary, negligible increased lifelong cancer risk could be expected for Pb contents of these market leafy vegetables for the exposed population in Johannesburg. OMUW leaves exhibited the greatest overall cancer risk, followed by OMW, SMW and VMW vegetables.

Estimated weekly intake of Al through OMUW leaves exceeded the TWI level of 1 mg kg^‒1^ bw week^‒1^ recommended by EFSA, but remained below the PTWI level of 2 mg kg^‒1^ bw week^‒1^ set by the JECFA (Fig. 2c). Across all washed leafy vegetable groups, EWI of Al stayed below the lower limit of TWI (Fig. 2c). Thus, Al contents of OMUW leaves may exert negative effects on the health of consumers. The weekly intake of Hg through unwashed and washed market leafy vegetables was under the JECFA recommended PTWI level of 0.004 mg kg^‒1^ bw week^‒1^ (Fig. 2d), implying no apparent adverse health consequences to consumers. Regarding both Al and Hg, EWI across the four leafy vegetable groups decreased in the following order: OMUW > OMW > VMW > SMW.

Trends in health consequences related to accumulated elements in leafy vegetables differed considerably between open-air markets and large stores in Johannesburg. Leafy vegetables from open-air markets presented the highest potential non-carcinogenic risks due to lifelong consumption of multiple individual toxic elements (Cd, Cr, As and Ni) through unwashed leaves and collectively all elements through unwashed and washed leaves. Furthermore, health issues triggered by Al toxicity, including impaired brain function and disorders associated with the skeletal and nervous systems, should not be disregarded as a result of regular consumption of unwashed OM vegetables (Mandal & Kaur, 2020). On the other hand, far lower non-carcinogenic risks linked to concurrent exposure to all elements could be expected for washed leafy vegetables from supermarkets and vegetable markets. Studies conducted in South Africa have reported unacceptable HI values for various fruits and vegetables from markets (Gupta et al., 2018; Nuapia et al., 2018).

Increased lifelong cancer risk of Cd and As through consumption of unwashed and washed leafy vegetables from open-air markets pose additional threats to the health of consumers. Compared to vegetables from open-air markets, much less but substantial cancer risk of Cd and As contents of leafy vegetables from supermarkets and vegetable markets may be expected. Hence, a greater cumulative health risk due to dietary intake of these leafy vegetables from different markets is probable over one’s lifetime.

This Johannesburg study highlighted some of the highly hazardous elements to human health, i.e. Cd, As, Cr and Ni, as the elements of greatest concern, a fact well-documented for vegetables from polluted urban regions worldwide (Alsafran et al., 2021; Gupta et al., 2018; Kananke et al., 2014). These elements therefore warrant additional attention in the study region. Due to relatively higher bioavailability and greater absorption through both roots and leaves, elevated accumulation of Cd in urban agricultural products and associated human health risks have been documented globally (Huang et al., 2017; Khan et al., 2020; Shahid et al., 2017). The negative impact of excess Cd ingestion could span from Itai-Itai disease, diabetes, renal diseases, cardiac arrest and stroke to cancer associated with the breast, oesophagus, lungs, intestine, stomach, prostate and testes (Kamunda et al., 2016; Carver & Gallichio, 2017). Likewise, high quantities of As intake may result in cancer of the bladder, lungs, and skin and can trigger various health issues such as nausea, vomiting, diarrhoea, skin ailments, immunological, cardiovascular and respiratory diseases, and kidney and liver damage (Kamunda et al., 2016; Carver and Gallichio 2017). For both Cd and As, intake of contaminated food has been identified as one of the major human exposure pathways (CAC, 2014; Carver and Gallichio 2017; Huang et al., 2017).

Cr generally shows a low root-to-shoot transfer rate. However, anthropogenic activity generated Cr exhibits increased bioavailability, leading to enhanced plant uptake and accumulation (Adhikari et al., 2022). Although Cr is recognized as a trace nutrient for humans, dermal and respiratory ailments and kidney and liver diseases are among the commonly observed toxicological effects of ingestion of high quantities of Cr (Kamunda et al., 2016; Khan et al., 2020). Unlike Cr, Ni shows higher mobility in the soil and within plant systems and tends to accumulate more in above-ground parts, especially leaves (Shahzad et al., 2018). Although Ni is required in trace amounts for human well-being, excess ingestion has been associated with problems related to blood pressure and nervous system and cardiovascular disorders (Lokeshappa et al., 2012).

This study validates a considerable reduction in both non-carcinogenic risks and cancer risks by washing leafy vegetables from roadside open-air markets with clean water. Thus, it is vital to raise awareness among urban populations about the potential adverse effects of consuming leafy vegetables exposed to hazardous deposition and the necessity of washing these vegetables before food preparation. The issue of dust deposition on vegetables should not be overlooked, especially considering climate change predictions indicating a decrease in rainfall and an increase in temperature in arid and semi-arid regions in South Africa and other countries around the world (Nhemachena et al., 2020). Consequently, drier conditions would accelerate emission and deposition of hazardous dust and resuspension of soil particles, as well as retention and transportation of particles through air currents would be prolonged. These effects could be notably pronounced in mining regions worldwide (Csavina et al., 2012). At the same time, a decrease in precipitation would lead to water scarcity and a reduction in water availability. Implementing effective dust pollution management and abatement measures in polluted urban regions in South Africa and elsewhere is therefore urgent.

## Conclusions

This study presents a comparative report on accumulation of selected elements and associated human health risks of leafy vegetables from roadside open-air markets and large stores (supermarkets and vegetable markets) in Johannesburg, South Africa. Findings revealed that unwashed leafy vegetables from roadside open-air markets not only had the highest concentrations of all evaluated elements, but also the sources of accumulated highly hazardous elements, including Cd, Ni, Cr, Hg and Pb, were primarily anthropogenic. Consequently, trends in non-carcinogenic risks related to individual elements varied mainly between unwashed leaves from open-air markets (with high risks associated with Cd, Cr, As and Ni) and washed leaves from all markets (that showed no potential health risk from any one element). In terms of health risks linked to all elements (presented as HI), leafy vegetables were largely differentiated between the open-air markets (for which HQ of Cd was the largest contributor to HI) and big stores (with HQ of Cr as the biggest contributor). Taken together, the non-carcinogenic risks associated with various individuals and all elements, along with high cancer risks of Cd and As, raise serious concerns regarding the regular consumption of unwashed and washed leafy vegetables from open-air markets. In contrast, far lower HI indicating non-carcinogenic risks associated with exposure to all elements and much less cancer risk of Cd and As suggest better suitability of these leafy vegetables from large stores for lifelong consumption. Hence, residents who primarily rely on open-air markets for their daily vegetable supplies could face substantially higher health risks compared to customers of large stores in Johannesburg. Therefore, deposition played a crucial role in driving accumulation of elements and subsequent human health risks of leafy vegetables from open-air markets. Thus, implementation of practical dust deposition prevention measures, such as covering displayed crops, placing barriers and planting trees between shops and roads, is urgent to reduce dust exposure to vegetables in open-air markets. Continuous monitoring and research efforts are essential for a better understanding of and developing strategies to manage contamination of vegetables by hazardous element exposures in polluted environments. This becomes particularly critical in light of predicted climate changes, specifically an increase in temperature and a reduction in precipitation, which will accelerate the generation and dispersion of hazardous dust.

## Data Availability

Not applicable.
